# Thymic Regulatory T Cell Development: Role of Signalling Pathways and Transcription Factors

**DOI:** 10.1155/2013/617595

**Published:** 2013-09-26

**Authors:** Mark Engel, Tom Sidwell, Ajithkumar Vasanthakumar, George Grigoriadis, Ashish Banerjee

**Affiliations:** ^1^Centre for Inflammatory Diseases, Monash Medical Centre, Southern Clinical School, Monash University, Clayton, VIC 3168, Australia; ^2^Molecular Immunology Division, Walter and Eliza Hall Institute of Medical Research, Melbourne, VIC 3052, Australia; ^3^Department of Clinical Haematology, Central Clinical School, Melbourne, VIC 3000, Australia

## Abstract

Regulatory T cells (Tregs) are a subset of CD4 T cells that are key mediators of immune tolerance. Most Tregs develop in the thymus. In this review we summarise recent findings on the role of diverse signalling pathways and downstream transcription factors in thymic Treg development.

## 1. Regulatory T Cells: Where Do They Come from and Why Do We Need Them?

The primary function of the mature T cell population is to mediate immune responses against a diverse array of foreign antigens, while remaining unresponsive to self-antigens. While the diversity of the T cell population is generated by the semirandom rearrangement of T cell receptor (*αβ*TCR) genes during development in the thymus, tolerance towards self is enforced both in the thymus and periphery known as central and peripheral tolerance, respectively. Fine-tuning the TCR repertoire of the T cell pool during intrathymic development is achieved via two ways: the delivery of survival signals to those cells that successfully ligate their TCRs to MHC molecules loaded with self-peptides with low to moderate affinity (positive selection) and the induction of apoptosis in those that recognise the same ligands strongly enough to be potential mediators of autoimmune disease (negative selection). The latter is aided by the transcription factor Aire (autoimmune regulator) which facilitates the thymic “promiscuous expression” of a diverse array of tissue specific antigens. The remaining cells that fail to recognise self-peptide MHC complexes, such as those which fail to express a mature *αβ*TCR, undergo time-dependent apoptosis, termed “death by neglect” [1–3]. The ideal outcome of this stringent and inefficient process of thymic education is a mature T cell repertoire whose TCRs successfully bind self-MHC molecules, while remaining unresponsive to autoantigens. Unfortunately, “central tolerance” to self is incomplete even in healthy mice and humans, in which peripheral T cells with potentially autoreactive TCRs can be found. These cells are kept unresponsive by immune cells whose function is to execute “peripheral” tolerance, with the best studied population being the Foxp3^+^ CD4^+^ regulatory T (Treg) cells [4, 5].

Treg cells were first identified as T cells that express high levels of the surface molecule CD25 and are capable of suppressing autoimmune reactions [4, 6]. Subsequent identification of various surface markers such as the glucocorticoid-induced TNF receptor (GITR), the cytotoxic T lymphocyte antigen 4 (CTLA4), and notably the forkhead box transcription factor Foxp3, which is a Treg-lineage specific transcription factor in mice, allowed further characterisation of these cells [7–9]. Most Tregs develop in the thymus (tTregs) [10], with “induced” or “adaptive” Tregs (pTregs for peripherally derived Tregs) generated via diverse mechanisms from naive CD4 T cells [11] also contributing to the peripheral Treg pool. How tTregs, which reach functional maturity within the thymus, are generated has been the subject of considerable scientific debate. Early work with double-transgenic mice expressing a monoclonal TCR and cognate neoautoantigen within the thymus showed a definite skewing of the mature thymic CD4SP population towards a CD25^+^ Foxp3^+^ regulatory phenotype [12–14], in a TCR affinity-dependent manner [13]. These data and similar subsequent work were the basis of the prevailing hypothesis that developing thymocytes are directed down the tTreg lineage by high-affinity TCR interactions with thymic peptide: MHC complexes [15]. 

## 2. Role of TCR, CD28, and IL-2R Signalling in Thymic Treg Development

**Figure 1:**
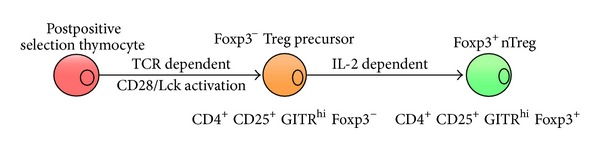
The stages of tTreg development. Thymic Treg development is achieved in two stages. Initially, postpositive selected thymocytes undergo TCR and CD28 dependent maturation into a Foxp3^−^ tTreg precursor population. A subsequent IL-2 dependent step leads to the development of Foxp3 expressing mature tTregs.

While there are a number of unreconciled observations in the literature, objective data regarding discrete developmental steps prior to the appearance of Foxp3-expressing mature tTreg cells have emerged. A recent report has identified at least two distinct phases in tTreg development, based upon differential dependence on TCR signals [16]. They identified a Foxp3^−^ population (tTreg precursors) and demonstrated that it was enriched for tTreg-specific TCRs—indicating prior selection on the basis of TCR—and required only common-*γ* chain (*γ*_*c*_) cytokines (predominantly IL-2) to differentiate into mature, Foxp3-expressing tTreg cells. Based on these observations the “two-step” model of tTreg cell development was proposed. In the first step, thymocytes undergo TCR and coreceptor dependent selection that gives rise to the CD4^+^CD25^+^GITR^hi^ Foxp3^−^ tTreg precursors [17, 18]. A subsequent TCR-independent IL-2/IL-15 dependent step results in Foxp3 expression marking the differentiation of tTreg precursors into tTregs [16, 19]. This two-step model is the currently prevailing framework through which thymic tTreg development is analysed (Figure 1).

TCR ligation eventually leads to activation of the transcription factors NF-AT and NF-*κ*B [20, 21]. The activation of NF-AT depends on its dephosphorylation by the Ca^2+^ dependent phosphatase Calcineurin [22]. Although, both NF-AT and Calcineurin pathways have been implicated in tTreg development, their precise role in this process is controversial [23–25]. The redundant function of different NF-AT isoforms expressed in T lineage cells has made it difficult to ascertain the role of this group of transcription factors in tTreg development. NF-AT is required for the induction of Foxp3 transcription *in vitro* [26, 27]. However, thymic regulatory T cell development and function are relatively normal in NF-ATc1/NF-ATc4 double deficient mice [28], with the suggestion that NF-ATc2 may compensate for the lack of NF-ATc1 and NF-ATc4 in these animals. Notably, mice deficient for NF-ATc2 and NF-ATc3 also exhibit normal tTreg development [29]. A severe defect in CD4 SP development due to a lack of NF-AT and ERK activation pose significant challenges to the study of Treg development in CalcinuerinB1 deficient mice [30–32]. Therefore, based on current experimental evidence, the Calcineurin/NF-AT pathway appears to be largely redundant in Treg development in the thymus. The apparent inconsistency between the requirement of NF-AT for Foxp3 expression *in vitro* (discussed later) and redundant function in tTreg development is further complicated by the finding that Calcineurin is not required for constitutive nuclear localisation of NF-AT in Tregs [33]. 

A number of reports have described a role for NF-*κ*B in tTreg development [23, 34–37]. TCR dependent activation of NF-*κ*B is mediated by a scaffold protein CARMA1, which belongs to the family of membrane associated guanylate kinases (MAGUK) [38–40]. TCR ligation leads to the calcium dependent activation of PKC*θ*, which in turn phosphorylates CARMA1 resulting in the recruitment of Bcl10 and MALT1. The CARMA1/Bcl10/MALT1 trimer, also known as the CBM complex, acts as a signalling platform that recruits and activates the IKK (I*κ*B kinase) complex, ultimately resulting in NF-*κ*B activation [41–43]. The Rel/NF-*κ*B family of transcription factors is comprised of 5 members, NF-*κ*B1, NF-*κ*B2, RelA, RelB, and c-Rel. Homo- and heterodimers of Rel/NF-*κ*B proteins exist as latent complexes bound to I*κ*B (inhibitor of *κ*B) proteins in the cytosol. Signals emanating from diverse cell surface receptors including the TCR result in IKK dependent phosphorylation of I*κ*B, targeting it for ubiquitin-dependent proteasome-mediated degradation, thereby allowing NF-*κ*B dimers to enter the nucleus and bind to decameric *κ*B sites on regulatory elements of various genes [44, 45]. NF-*κ*B regulated gene expression impinges on diverse processes downstream of the TCR including T cell development, survival, and cytokine production [46]. Given such functional diversity displayed by NF-*κ*B in CD4 T cell development/activation, it is not surprising that it plays a role in tTreg development. Mice deficient in PKC*θ* [23, 37] or CARMA1 [34, 35] or those that lack Bcl10 [37] exhibit significant reduction in tTregs. A conditional deletion of *Ikkβ* in DP thymocytes also results in loss of tTregs [36]. tTreg development has also been studied in some individual *NF-*κ*B* knockout mice. While tTreg numbers are relatively normal in *NF-*κ*B1* ^−/−^ mice [18], the absence of RelA results in a 2-fold reduction in thymic CD4^+^CD25^+^Foxp3^+^ cells [47]. Of all the NF-*κ*B family members, c-Rel is the most important player in tTreg development with a ~6–10 fold reduction in tTreg numbers in *C-rel* ^−/−^ mice [18, 47–51]. It is important to note that like CARMA1, c-Rel also plays a nonredundant cell intrinsic role in the development of tTreg precursors [18, 35, 52]. It is believed that both CARMA1 and c-Rel deficient Treg precursors have impaired IL-2R signaling [53, 54] and as a consequence fail to efficiently differentiate into Foxp3^+^ tTregs in response to IL-2 [52].

Signalling downstream of CD28 synergises with TCR signals to promote optimal Treg development [55]. Although the role of CD28 signalling in peripheral homeostasis and survival of Tregs is well appreciated [56–58], the precise mechanism by which costimulatory signals regulate tTreg development is poorly understood. Interaction of B7 with CD28 is believed to strengthen contact between the antigen presenting cell and developing thymocyte thereby promoting survival of the latter via IL-2 production and upregulation of prosurvival Bcl_XL_ [59–61]. Both CD28 and B7 knockout mice have significant reduction in thymic Treg numbers [18, 55, 57, 58]. An elegant study by Tai et al. described a PYAP motif (aa 187–190) in the cytoplasmic tail of CD28 that is required for tTreg development. Apart from binding the tyrosine kinase Lck, this motif is also required for IL-2 production. Although transgenic overexpression of a wild-type CD28 transgene significantly rescued tTreg numbers in Cd*28* ^−/−^ mice, a mutant CD28 transgene where both Prolines in the PYAP motif were substituted with Alanine failed to do so [55]. Recent work has demonstrated that CD28 is also required for the generation of tTreg precursors. Using mixed bone marrow chimeras, a cell intrinsic role of CD28/Lck in development of tTreg precursors was described [18]. The requirement of Lck in CD28 dependent tTreg development is not restricted to generation of tTreg precursors as Lck activation is also required for expression and stabilisation of Foxp3 message [62]. Interestingly, IL-2 deficiency in mice has little to no impact on tTreg numbers [63, 64]. However, IL-2 plays an important role in the survival of mature Tregs [63, 64]. 

## 3. Transcriptional Control of *foxp3 * in Thymic Tregs

Humans with a mutation in their *foxp3* gene suffer from a spontaneous inflammatory disease called IPEX [65–67] (Immune dysregulation Polyendocrinopathy Enteropathy X linked syndrome). Additionally, a naturally occurring *Foxp3* mutation in mice (*Scurfy*) results in a severe T cell dependent multiorgan autoimmune and inflammatory disease [68, 69]. These findings necessitated closer examination of how the expression of this crucial transcription factor is controlled.

The architecture of the *Foxp3* promoter along with its downstream *cis* regulatory elements determines Treg-specific expression and maintenance of Foxp3. The promoter of *Foxp3* is located ~6.6 kb upstream of its translational start site and has weak transcriptional activity [70] which is enhanced by the binding of several transcription factors (TFs) to the *cis* regulatory elements located in the introns. Two evolutionarily conserved noncoding sequences (CNS), *Cns1* and *Cns2,* located in the first intron between the two 5′UTR exons act as enhancer elements [70]. Additionally, an enhancer element, *Cns3*, located in the third intron immediately after the first coding exon serves an indispensable role in the induction of *Foxp3* expression during thymic Treg development [50]. The importance of these CNSs in *Foxp3* expression and Treg development was demonstrated by systematic deletion of these *cis *acting elements in mice [50]. *Cns1* was shown to be dispensable for tTreg development but required for TGF*β* dependent conversion of naive CD4^+^ T cells into Tregs. *Cns2* ^−/−^ mice did not display major defects in thymic Treg development. However,* Cns2* deletion adversely affected the stability of Foxp3 expression. In contrast, deletion of *Cns3* resulted in a significant reduction in thymic Treg numbers. TCR, CD28, and IL-2 dependent signals activate and recruit TFs to the promoter and *Cns2* and *Cns3* enhancer elements to initiate *Foxp3* transcription and maintain stable expression of Foxp3 in tTregs. These enhancer regions are also amenable to epigenetic modifications, which serve as an additional layer of transcriptional control imposed on *Foxp3* expression.

## 4. Differential DNA Methylation Regulates *foxp3* Transcription 

The *Cns2 *element encompasses a CpG island that is hypomethylated only in thymic Tregs [48]. TCR signals result in *Cns2* demethylation rendering this element permissive for the binding of transcription factors induced downstream of TCR, CD28, and the IL-2R [70–73]. Pharmacological inhibition of DNA methylation resulted in *Foxp3* induction in conventional CD4^+^ T cells [74] supporting the notion that methylation of *Cns2* is prohibitive for *Foxp3* expression. Conversely, reducing the expression of the DNA methyltransferase, Dnmt1, using shRNA mediated knockdown also resulted in *Foxp3* expression, reinforcing the importance of *Cns2* demethylation in thymic Treg development [70]. In addition to the induction of *Foxp3* transcription, demethylation of *Cns2* is also required for stable Foxp3 expression [50]. Although forced demethylation of *Cns2* is known to initiate *Foxp3* transcription, the exact TCR dependent mechanism(s) that demethylates *Cns2* during tTreg development is unclear. It is important to appreciate that the exact role of *Cns2* is hard to define in the absence of further knowledge about factors that bind this region. The lack of a phenotype in *Cns2* ^−/−^ mice may mean that Foxp3 expression proceeds unimpeded because a negative regulatory region (*Cns2*) is no longer there. In other words, the function of *Cns2* demethylating factors is made redundant in *Cns2* ^−/−^ mice.

Like *Cns2*, the *Foxp3* promoter region has interspersed CpG motifs the methylation status of which is critical for the initiation of transcription. The *Foxp3* promoter region is completely methylated in naive CD4 T cells and partially methylated in Tregs [70, 74]. However, unlike *Cns2*, promoter demethylation increases as Tregs mature and migrate to the periphery [75, 76]. Like *Cns2*, demethylation of the promoter is permissive for the binding of several TFs to initiate the expression of *Foxp3* in response to TCR, CD28, and IL-2R signals. Supporting this notion, a negative regulator of Treg development PIAS1 maintains the *Foxp3* promoter in its methylated state by binding to it and recruiting DNA methyltransferases and Heterochromatin protein 1 resulting in the formation of a “repressive complex” [77]. This “repressive complex” collapses upon TCR signalling and the promoter becomes accessible to TFs to participate in the initiation of transcription [77]. 

## 5. TCR- and IL-2-Induced Transcription Factors Induce *Foxp3* Expression in tTregs

Several TFs have been implicated as essential factors in Foxp3 expression. Primarily the NF-*κ*B family member, c-Rel, has been demonstrated to be important in *Foxp3* induction during tTreg development. The drastic reduction of tTreg numbers in *C-rel* ^−/−^ mice is linked to a defect in the induction of *Foxp3* expression [48]. To date, three different mechanisms have been proposed to explain the role of c-Rel in *Foxp3* induction. Given TCR signalling is responsible for *Cns2* demethylation with c-Rel being activated downstream of the TCR, it is possible that recruitment of c-Rel to *Cns2 *results in its demethylation [48]. This hypothesis stems from the finding that c-Rel can bind to the methylated *Cns2 *region in the *Foxp3* locus [48]. Therefore, it is plausible that demethylation of the *Cns2* region induced by the binding of c-Rel facilitates binding of other TFs resulting in the induction of *Foxp3* transcription. Yet another study elegantly demonstrated that c-Rel nucleated an “enhanceosome” comprising other TFs including p65 (RelA), pCREB, and NF-ATc2 at the *Foxp3* promoter [49]. The authors utilised *in vitro* differentiated Tregs (iTregs) to demonstrate that the recruitment of c-Rel and RelA to the *Foxp3* promoter shortly after TCR stimulation led to the formation of this “enhanceosome.” In the absence of c-Rel the enhanceosome failed to form suggesting the central role of c-Rel in this process. Finally, Zheng et al. demonstrated the *in vitro* binding of c-Rel to a CD28 response element located in the *Cns3* region [50]. A similar reduction in tTregs observed in both *C-rel* ^−/−^ and* Cns3* ^−/−^ mice supports the model whereby c-Rel binding to *Cns3* results in thymic Foxp3 expression. Given *Cns3* is permissive from the DP stage to tTregs, c-Rel can potentially access *Cns3* regardless of the *Cns2* methylation status. The binding of c-Rel to *Cns3* is also believed to induce chromatin remodelling at the adjacent *Cns2* element. The resultant *Cns3*-c-Rel-induced permissive status of *Cns2* can be utilised by other TFs for the initiation of *Foxp3* transcription. 

**Figure 2:**
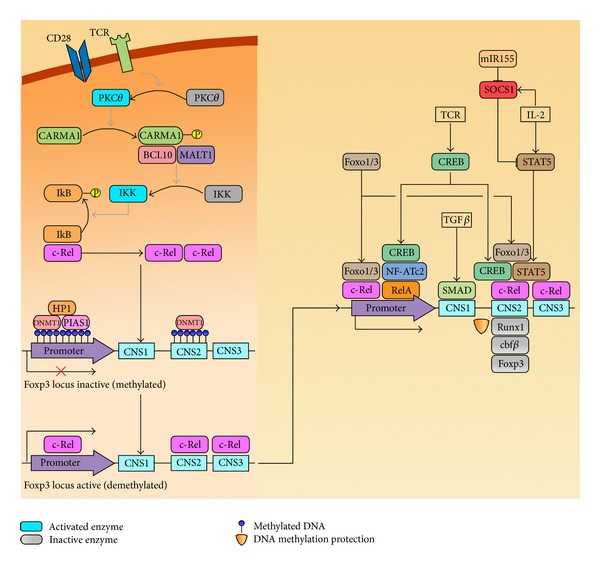
Transcription factors in tTreg development. Once engaged, cell surface receptors initiate c-Rel activation and nuclear entry. c-Rel binding to the *Foxp3* promoter and *Cns* elements promotes epigenetic changes, including demethylation and chromatin remodelling (not pictured) of the *Foxp3* locus. Several other transcription factors cooperate with c-Rel to initiate and preserve the stable expression of *Foxp3* in tTregs.

The demethylated *Cns2* region also recruits cyclic-AMP response element binding protein (CREB)/activating transcription factor (ATF) in response to TCR signals [70]. Another group of proteins known to induce Foxp3 expression in tTregs belong to the Foxo family of TFs and include Foxo1 and Foxo3 that bind to the promoter and *Cns2* elements to induce *Foxp3* expression. [78]. TCR and CD28 stimulation results in activation and nuclear entry of c-Rel. In contrast, Foxo protein that are constitutively present in the nucleus are phosphorylated and inactivated by the PI3K/Akt signalling axis downstream of TCR. Therefore it is possible that Foxo proteins and c-Rel bind sequentially to the *Foxp3* locus [78]. In addition to these transcription factors, IL-2-induced STAT5 also has a prominent role in controlling *Foxp3* expression [71–73]. IL-2 signalling induces increased levels of SOCS1, which negatively regulates STAT5 resulting in reduced *Foxp3* expression. Therefore, efficient expression of *Foxp3* requires suppression of SOCS1 levels in Tregs. The microRNA mIR155 targets *Socs1* mRNA in Tregs and consequently maintains high levels of STAT5 expression [79]. Deletion of *Mir155* in mice has been reported to result in upregulation of SOCS1 and subsequent inhibition of STAT5 mediated *Foxp3* expression in tTregs [79, 80]. Sustained or stable expression of Foxp3 which is critical for tTreg-lineage stability is maintained by the TFs, Runx1, and Cbf*β*. These TFs form a trimeric complex with Foxp3 and bind *Cns2* establishing a feed forward loop ensuring stable Foxp3 expression [50]. Given these findings, we propose that c-Rel, known as the “pioneer TF,” is the first TF to bind to the *Foxp3* promoter, *Cns3, and Cns2* following TCR ligation. The binding of c-Rel to *Cns3* and methylated *Cns2* results in demethylation of *Cns2* facilitating subsequent recruitment of Foxo1 and Foxo3. Phospho-STAT5 induced by IL-2 follows soon after ultimately resulting in *Foxp3* transcription. It is important to note that once demethylated, the *Cns2* CpG region is protected from methylating enzymes by the Foxp3-Runx1-Cbf*β* complex [50]. In summary, we propose that the TCR-/IL-2-induced transcriptional activation of *Foxp3* is initiated by c-Rel, which is then maintained by sequential binding of other TFs including Foxp3 itself (Figure 2).

## 6. Concluding Remarks

Since their discovery, regulatory T cells have been at the forefront of immunology research. Although we have made significant progress towards the understanding of their development and function a lot needs to be done to apply these findings in the clinic. Although a number of publications have reported modulation of Treg numbers and/or function in diverse disease, very little is known about the real impact of Treg modulation in disease onset and progression. It is critical to acknowledge that Tregs can be both beneficial and deleterious depending on the disease and stage. Therefore we believe that a careful analysis of Treg function in individual diseases is needed to meaningfully apply Treg based therapies in the clinic.
